# Update: Rationale and design of the *So*dium *L*owering *I*n *D*ialysate (SoLID) trial: a randomised controlled trial of low versus standard dialysate sodium concentration during hemodialysis for regression of left ventricular mass

**DOI:** 10.1186/s12882-015-0080-y

**Published:** 2015-08-01

**Authors:** Joanna Leigh Dunlop, Alain Charles Vandal, Janak Rashme de Zoysa, Ruvin Sampath Gabriel, Imad Adbi Haloob, Christopher John Hood, Philip James Matheson, David Owen Ross McGregor, Kannaiyan Samuel Rabindranath, David John Semple, Mark Roger Marshall

**Affiliations:** Auckland Clinical School, Faculty of Medical and Health Sciences, University of Auckland, Private Bag 93311, Otahuhu, Auckland 1640 New Zealand; Department of Renal Medicine, Middlemore Hospital, Counties Manukau District Health Board, Private Bag 93311, Otahuhu, Auckland 1640 New Zealand; Faculty of Health and Environmental Sciences, Auckland University of Technology, North Shore Campus, Private Bag 92006, Auckland, 1142 New Zealand; Ko Awatea, Middlemore Hospital, Counties Manukau District Health Board, Private Bag 93311, Otahuhu, Auckland 1640 New Zealand; Renal Service, North Shore Hospital, Waitemata District Health Board, Private Bag 93503, Takapuna, Auckland 0740 New Zealand; Department of Cardiology, Middlemore Hospital, Counties Manukau District Health Board, Private Bag 93311, Otahuhu, Auckland 1640 New Zealand; Bathurst Health Service, New South Wales, Australia; Department of Nephrology, Wellington Hospital, Capital & Coast District Health Board, Private Bag 7902, Wellington South, New Zealand; Department of Nephrology, Christchurch Hospital, Canterbury District Health Board, Private Bag 4710, Christchurch, New Zealand; Department of Renal Medicine, Waikato Hospital, Waikato District Health Board, Private Bag 3200, Hamilton, 3240 New Zealand; Department of Renal Medicine, Auckland City Hospital, Auckland District Health Board, Private Bag 92024, Auckland, Auckland 0740 New Zealand; Medical Affairs - Renal (Asia Pacific), Baxter Healthcare, P.O. Box 14062, Panmure, Auckland 1741 New Zealand

## Abstract

After the publication of our paper Dunlop et al. “Rationale and design of the Sodium Lowering In Dialysate (SoLID) trial: a randomised controlled trial of low versus standard dialysate sodium concentration during hemodialysis for regression of left ventricular mass”, we became aware of further data correlating left ventricular (LV) mass index at baseline and their correpsonding mass at 12 months, using cardiac margnetic resonanace imaging (MRI) in patients on hemodialysis. The original published sample size for the SoLID trial of 118 was a conservative estimate, calculated using analysis of covariance and a within person Pearson’s correlation for LV mass index of 0.75. New data communicated to the SoLID trial group has resulted in re-calcuation of the sample size, based upon a within person Pearson’s correlation of 0.8 but otherwise unchanged assumptions. As a result, the SoLID trial will now recruit 96 participants.

## Update

After the publication of our paper Dunlop et al. “Rationale and design of the Sodium Lowering In Dialysate (SoLID) trial: a randomised controlled trial of low versus standard dialysate sodium concentration during hemodialysis for regression of left ventricular mass” [1], we became aware of further data correlating left ventricular (LV) mass index at baseline and their correpsonding mass at 12 months, using cardiac margnetic resonanace imaging (MRI) in patients on hemodialysis.

The original published sample size for the SoLID trial of 118 was a conservative estimate, calculated using repeated-measures analysis of covariance (ANCOVA) and a within person Pearson’s correlation for LV mass index of 0.75. The assumption of a correlation of 0.75 was based on private communication from the Jardine group (private communication P Mark 8/2/2011) [2–4]: in a cohort of 59 patients of their patients with repeated measures of LVMI at least 6 months apart (using cardiac MRI), correlation was 0.87 (p < 0.001) with normally distributed data. Modelling these data, and allowing for 25 % for drop outs, it was determined that 59 participants would be enrolled in each arm (power 0.8, alpha 0.05).

New data communicated to the SoLID trial group has resulted in us re-calculating sample size for the SoLID trial (private communication C McIntyre and A Odudu 30/4/2014) [5]: in a cohort of 44 patients of their patients with repeated measures of LV mass index 12 months apart (using cardiac MRI), correlation was 0.81 (p < 0.001). In 21 of these patients, there was an intervention that might have modestly affected their LV mass index; however, restricting analysis to the 23 paired values in the control group provides a similar correlation of 0.83.

Based on the data above, the sample size has been recalculated using a within person correlation of 0.8, but otherwise unchanged assumptions. It is was determined that 48 participants will be enrolled in each arm. The change in sample size has been approved by the National (New Zealand) Multi-region Ethics Committee (IRB00004663) of the New Zealand Ministry of Health (IORG0000895), and the primary funding body for this study the Health Research Council of New Zealand.
